# A Scoping Review of How Financial Hardship Is Measured Among Older Adults in the United States

**DOI:** 10.1093/geroni/igaa057.348

**Published:** 2020-12-16

**Authors:** Reginald Tucker-Seeley, Ryan DoyLoo, Leora Steinberg

**Affiliations:** 1 University of Southern California, Los Angeles, California, United States; 2 Tucker-Seeley Research Laboratory, Malibu, California, United States

## Abstract

The association between socioeconomic status and health/healthcare related outcomes across the life course is well established; however, the specific mechanisms that underlie this complex association are not well understood. There have been calls in the health disparities literature for greater explication of the socioeconomic factors associated with differential outcomes for racial/ethnic minorities and socioeconomic groups. Recent research offers an expanded notion of socioeconomic circumstances by including indicators of financial hardship; however, there has been little conceptual and measurement clarity for gerontology research. To fill this gap, we conducted a scoping review of how financial hardship has been defined and measured in research with older adults. Using an adapted version of the Preferred Reporting Items for Systematic reviews and Meta-Analyses (PRISMA) protocol, N=25 articles were identified through the following indexes: PubMed, CINAHL, PsycInfo, Sociological Abstracts, and Embase. Articles were included if they were published after January 1, 2000, published in the United States, and conducted with adults aged 50 and older. Our study found neither a consistently used term for nor a definition of the financial hardship experience; however, two key domains were consistently measured across studies with older adults: food insecurity and medical expenses were measured in N=9 and N=13 studies, respectively. Greater conceptual and measurement clarity in research on financial hardship among older adults helps to unpack the complex ways in which socioeconomic circumstance are experienced, make comparisons across studies measuring the financial hardship experience, and to identify the specific aspects of financial hardship for intervention.

